# Health and disability assessment in individuals with hearing loss: a longitudinal study using WHODAS 2.0

**DOI:** 10.1590/2317-1782/e20250297en

**Published:** 2026-07-31

**Authors:** Karen Melissa Gonzaga dos Santos, Rogéria Cristina Toscano Dias, Jade Louise Alves Macedo Padilha Silva, Gizele Francisco Ferreira do Nascimento

**Affiliations:** 1 Residência Multiprofissional no Cuidado à Saúde da Pessoa com Deficiência, Instituto de Ensino e Pesquisa Alberto Santos Dumont – ISD - Macaíba (RN), Brasil.; 2 Programa Associado de Pós-graduação em Fonoaudiologia – PPgFon, Universidade Federal do Rio Grande do Norte – UFRN - Natal (RN), Brasil.

**Keywords:** Hearing, Hearing Loss, International Classification of Functioning, Disability and Health, Hearing Aids, Hearing Impairment Rehabilitation

## Abstract

**Purpose:**

To assess the self-perception of health and disability of individuals with bilateral hearing loss (HL) before and after one year of using hearing aids, using the World Health Organization Disability Assessment Schedule 2.0 (WHODAS 2.0).

**Methods:**

Longitudinal, observational and analytical-descriptive study, approved by the research ethics committee under opinion number 6.767.314, in which 15 individuals diagnosed with mild to severe hearing loss and wearing bilateral hearing aids (HA) were assessed. The WHODAS 2.0 was applied at the time of fitting and reapplied after one year of use. The mean scores in the six domains of the instrument (cognition, mobility, self-care, getting along, life activities and participation) were statistically analyzed.

**Results:**

A reduction in disability was observed after using HA, with an average reduction of 12% in WHODAS scores. Domains such as cognition, self-care, and participation showed statistical differences.

**Conclusion:**

WHODAS 2.0 proved effective in assessing and monitoring disability and its impact on the functioning of individuals with HL, being sensitive to changes promoted by the use of HA, despite the absence of statistical significance in some variables.

## INTRODUCTION

Hearing loss (HL) is one of the most prevalent and neglected sensory conditions of the 21st century, affecting more than 430 million people worldwide and representing one of the leading causes of disability, especially among adults and the elderly^(1)^. It is estimated that, starting at age 60, approximately 20% of individuals have some degree of HL, which often goes undiagnosed or is not treated properly^(2)^. The impact of HL extends beyond hearing itself and directly affects the quality of life, mental health, and social interactions of those affected^(3)^.

Hearing rehabilitation is considered an urgent demand for public health systems, given the context of global population aging, which is leading to a growing number of individuals with HL due to presbycusis. The use of hearing aids (HA) is considered one of the main therapeutic strategies to enhance auditory perception and improve communication. However, the effectiveness of HA extends beyond audiometric gains, as they have an impact on various aspects of daily life, such as autonomy, social participation, occupational performance, and emotional well-being^(3)^.

In order to assess the impact of these issues on an individual’s quality of life, it is necessary to adopt the biopsychosocial model, which views health as the result of the interaction between biological aspects and contextual, environmental, and personal factors^(4)^. In this scenario, the International Classification of Functioning, Disability, and Health (ICF) defines “functioning” as an overarching term for the positive aspects of interaction, while “disability” reflects the negative aspects, such as limitations and restrictions on participation^(5)^. In the context of audiology, the ICF allows functionality to be understood as an umbrella term for the positive aspects of hearing health, while disability reflects the challenges individuals face in their daily activities and social participation^(4)^.

This biopsychosocial perspective is essential for hearing rehabilitation to go beyond audiometric gain and focus on reducing the participation limitations experienced by the individual^(4,5)^. To quantify these parameters in a standardized manner, the World Health Organization Disability Assessment Schedule 2.0 (WHODAS 2.0) was developed as a tool directly based on the concepts and domains of the ICF, enabling the measurement of the level of perceived disability across six domains: cognition, mobility, self-care, getting along, life activities, and participation^(5)^.

The WHODAS 2.0 has been widely used in various populations, including individuals with chronic diseases, mental disorders, physical and neurological disabilities, and has demonstrated reliability, validity, and sensitivity to changes over time^(6,7)^. Despite this, its application in people with hearing impairments remains under-explored in the literature, mainly in studies that assess the impact of HA fitting on the perception of disability. Recent research suggests that the continuous use of conventional hearing devices, such as HA, may not only improve speech comprehension, but also reduce barriers to social participation, enhance self-confidence, and promote greater engagement in social and occupational activities^(8)^.

Given this, it is necessary to assess how health and disability profiles are affected by the use of HA, using tools such as the WHODAS 2.0, as it can provide clinical support for planning individual-centered interventions and allow objective measurement of hearing rehabilitation outcomes from a biopsychosocial perspective, in addition to contributing to the formulation of public policies that recognize the real impact of HL on people’s lives and guarantee effective interventions.

Thus, the present study aimed to assess the self-perceived health and disability of individuals diagnosed with bilateral hearing loss who use hearing aids, before and after one year of device use, utilizing the WHODAS 2.0 as a measurement tool.

## METHODS

This is a longitudinal, observational, and descriptive-analytical study, approved by the Research Ethics Committee under Opinion No. 6.767.314. The preliminary sample was obtained for convenience from patients followed by the Hearing Health Service at the Alberto Santos Dumont Institute of Education and Research, located in the rural area of the municipality of Macaíba, Rio Grande do Norte (Brazil). All individuals who agreed to participate in the study were informed of the study’s aims and signed the Informed Consent Form (ICF).

The study included 15 individuals diagnosed with bilateral HL, ranging from mild to severe, who were candidates for HA. The inclusion criteria were as follows: age 18 years or older, no other sensory or cognitive impairments that could interfere with the understanding of the instrument, and availability for longitudinal follow-up. Participants who used HA irregularly or had a historical record of discontinuity in audiological follow-up were excluded.

Data collection was conducted in person using the 36-item version of the WHODAS 2.0, administered by audiologists trained in the application of the instrument. The first assessment was conducted between January and December 2023, during the initial period of adjustment to the HA. The second assessment took place between January and December 2024, after approximately 12 months of regular use of the device. In addition, through a review of medical records, we collected information on the participants’ age, gender, type and degree of HL, educational level, and occupational status.

The WHODAS is a practical assessment developed by the WHO based on the ICF concept, designed to measure levels of health and disability in a standardized manner by assessing six functional domains, as follows: D1 – cognition (understanding and communicating); D2 – mobility (moving and getting around); D3 – self-care (hygiene, dressing, and eating); D4 – getting along; D5 – life activities; and D6 – participation in social and community activities. Each item on the tool is scored on a scale of 0 to 4, where “0” represents no difficulty and “4” represents extreme difficulty or disability. Scores were calculated by domain and also on an overall basis (total score and percentage of functional disability).

Statistical analysis was performed using the Statistical Package for the Social Sciences (SPSS), version 25.0. Initially, normality tests (Kolmogorov-Smirnov) were conducted, followed by descriptive statistics to characterize the sample. To perform a comparative analysis of the scores before and after HA use, we used Student’s t-test for paired samples. The Marginal Homogeneity test was used to assess changes in functional classification (mild, moderate, severe). Spearman’s correlation tests were also performed between age, hearing loss degree, and functional score, as well as one-way ANOVA for analysis of variance between groups. The level of significance adopted was p<0.05.

## RESULTS

The sample consisted of 15 participants, including 11 men (73.3%) and 4 women (26.7%), with a mean age of 63.8 years (±11.08).

Regarding the degree of HL, 2 participants had mild HL (13.3%), 8 had moderate HL (53.3%), and 5 had severe HL (33.3%). All participants were fitted bilaterally with digital HA, adjusted according to the pure-tone hearing thresholds obtained during the conventional audiological evaluation. Table 1 presents the sociodemographic and audiological data.

**Table 1:** Sample characteristics (n = 15)

**VARIABLE**	**VALUE**
**Gender - n (%)**	
Male	11 (73.3%)
Female	4 (26.7%)
**Age - mean ± SD**	63.8 ± 11.08
**Hearing loss - n (%)**	
Mild	2 (13.3%)
Moderate	8 (53.3%)
Severe	5 (33.3%)
**Educational level - n (%)**	
Incomplete elementary school	4 (26.67%)
Complete elementary school	8 (53.33%)
Incomplete high school	0 (0%)
Complete high school	3 (20%)
**Employment status - n (%)**	
Paid employment	7 (46.7%)
Retired	7 (46.7%)
Not employed	1 (6.6%)

Caption: n = number of individuals; SD = standard deviation

In terms of socio-educational background, it was observed that 53.3% of the sample had completed at least elementary school, while 26.7% had not completed elementary school and 20% had completed high school. Regarding employment status, there was an equal distribution between retired individuals (46.7%) and those engaged in some form of paid work (46.7%), while only one participant (6.6%) reported not currently being employed (Table 1).

In the initial assessment using the WHODAS 2.0, the total mean score was 1.87 (±0.65), corresponding to a mean percentage of 36.2%. After one year of regular HA use, a significant reduction in the total mean score was observed, which decreased to 1.21 (±0.29), with a mean percentage of 24.2%. This reduction corresponds to an average functional improvement of 12%, indicating a reduced impact of health limitations on daily activities.

Table 2 presents a detailed comparison of the means by domain before and after HA use. The domains of cognition (p=0.015), self-care (p=0.017), life activities (p=0.044), and participation (p=0.013) showed statistically significant differences between the assessed time points. The domains of mobility (p=0.142) and getting along (p=0.304), although showing a trend toward improvement, did not reach statistical significance.

**Table 2:** Mean WHODAS 2.0 scores at the start of HA fitting and after one year of use (n = 15)

**VARIABLE**	**VALUE**	**VALUE**	***p value***
	**baseline**	**after 1 year**	
**WHODAS 2.0 - mean ± SD**	1.87 ± 0.65	1.21 ± 0.29	0.014^*^
Domain 1 - Cognitive	1.94 ± 0.65	1.34 ± 0.44	0.015*
Domain 2 - Mobility	1.93 ± 0.95	1.46 ± 0.63	0.142
Domain 3 - Self-care	1.4 ± 0.51	1.03 ± 0.87	0.017*
Domain 4 - Getting along	1.72 ± 0.77	1.52 ± 0.36	0.304
Domain 5 - Work/household activities	1.64 ± 0.89	1.10 ± 0.18	0.044*
Domain 6 - Participation	2.12 ± 0.78	1.46 ± 0.62	0.013*
**Total percentage - mean ± SD**	36.2 ± 11.14	24.2 ± 5.8	0.017*
**Degree of disability - n (%)**			0.083
Mild	7 (46.7%)	11 (73.3%)	
Moderate	6 (40%)	4 (26.7%)	
Severe	2 (13.3%)	0 (0.0%)	

* p < 0.05

**Caption:** WHODAS 2.0 = World Health Organization Disability Assessment Schedule 2.0; n = number of individuals; SD = standard deviation

In addition, a change was observed in the participants’ functional profile: the number of individuals with mild disability increased from 7 (46.7%) to 11 (73.3%), while moderate cases decreased from 6 (40%) to 4 (26.7%) and severe cases were no longer observed. Despite the evident clinical improvement, the Marginal Homogeneity test did not indicate a statistical difference (p=0.083), possibly due to the small sample size.

Spearman’s correlation analyses revealed no significant association between functional scores and age (p=0.380) or degree of hearing loss (p>0.05). The ANOVA test also identified no significant variability in WHODAS scores based on these variables, suggesting that functional improvement occurred independently of the audiological profile.

## DISCUSSION

The results of this study show that consistent use of the HA led to a measurable improvement in the functional ability of individuals with HL, as evidenced by lower WHODAS 2.0 scores after one year of follow-up. Although some analyses did not reach statistical significance, the results indicate important clinical effects, particularly in the areas of cognition, self-care, life activities, and participation — areas that are fundamental to maintaining independence and quality of life.

These findings reinforce the existing literature highlighting the functional impact of hearing loss across various aspects of life. The presence of hearing loss is associated with cognitive decline, an increased risk of depression, impaired interpersonal communication, social isolation, and a reduced ability to perform instrumental activities of daily living. In this regard, hearing amplification, by partially restoring sound perception, tends to alleviate the cognitive overload caused by hearing difficulties and to promote the individual’s social reintegration^(3,8,9)^.

The domain of participation proved to be one of the areas most benefited by the use of HA, with a significant reduction in limitation scores. The literature suggests that participation in social and community activities is one of the first areas affected by HL, often due to communication anxiety and the stigma associated with the use of hearing devices^(10)^. With regular use of HA, users tend to regain some of their confidence and ability to engage in social situations, which can have positive effects on mental health and functional self-perception^(11-13)^.

Another finding was an improvement in cognitive function, which supports recent research indicating a bidirectional relationship between HL and cognitive decline^(12,13)^. The “increased cognitive load” hypothesis suggests that, in the presence of hearing loss, the brain must redirect cognitive resources to compensate for sensory loss, at the expense of other functions such as memory, attention, and planning^(14)^. The use of hearing aids can reduce this cognitive overload and preserve executive functions, especially in older adults.

In addition to the significant improvements already anticipated in the domains of Cognition and Participation, this study also found a statistically significant reduction in disability in the domains of Self-Care and Life Activities. The improvement in the “Life Activities” domain is likely linked to the reduced cognitive load provided by the HA. Since the effort required to listen decreases, the individual is able to allocate more attentional resources to performing occupational and daily tasks^(15)^. As for the significant improvement in self-care, it can be inferred that hearing rehabilitation leads to improved environmental perception and self-confidence in communication, which may result in an increase in the individual’s overall autonomy, as they begin to feel more confident in managing their personal activities independently^(16)^.

The sample characteristics revealed a profile predominantly composed of individuals with low levels of education and a homogeneous occupational status. In addition, the higher proportion of male participants reflects the profile of the service’s demand where the data were collected, as this was a convenience sample. In the biopsychosocial model, gender is a significant personal factor that, along with educational attainment, can influence understanding of health, adherence to treatment, and self-perception of disability. These findings are significant, as limited access to education is, for the most part, a factor that can affect the perception of disability^(11,12)^.

Furthermore, the employment situation, as reflected by the large number of individuals working, may be correlated with improvements in the “Life Activities” domain, suggesting that hearing rehabilitation has a direct impact on maintaining productivity. On the other hand, the number of retired participants may also be contributing to improvements in the domains of “Participation” and “Cognition,” since HA plays a key role in promoting active aging and quality of life^(13,16)^.

The lack of significance in the domains of Mobility and Getting Along may have multiple explanations. Mobility, although it may be indirectly influenced by hearing — through balance and spatial orientation — is not directly affected by sound amplification in individuals without otoneurological comorbidities. Getting Along, on the other hand, involves multiple contextual, emotional, and cultural factors, and changing these may require interventions that complement the use of HA, such as psychological support.

WHODAS 2.0 proved to be a sensitive tool for capturing functional changes resulting from hearing rehabilitation, even in a small sample. Its ICF-based approach allows for an integrated, user-centered perspective, aligned with the principles of current audiology practice^(17)^. Previous research has confirmed its effectiveness among older adults, people with disabilities, and those with chronic conditions^(13)^ and, in the present study, its applicability was observed in users of HA.

However, the limitations of this study should be noted, such as the small sample size, which limited the feasibility of more in-depth statistical analyses and may have contributed to the lack of statistical significance in some comparisons, and the sampling method — convenience sampling — which resulted in a gender imbalance, given that gender is considered a relevant personal factor in the biopsychosocial model. In addition, data regarding participants’ income were not discussed, limiting the analysis of differences across socioeconomic profiles. Consequently, it was not possible to explore how disability was perceived across the groups.

Nevertheless, the observed results have significant practical implications. WHODAS 2.0 can be incorporated into clinical practice as a tool for assessing and monitoring functional status over time in individuals with HL, helping to establish personalized treatment goals and evaluate the impact of hearing rehabilitation from multiple perspectives.

## CONCLUSION

This study demonstrated that consistent use of HA is associated with functional improvement in individuals with hearing loss, particularly in the areas of cognition, self-care, activities of daily living, and social participation. WHODAS 2.0 has proven effective in measuring these changes over time, reinforcing its value as a clinical monitoring tool.

Although some analyses did not reach statistical significance, a positive clinical trend was observed, highlighting the importance of assessing functionality in audiology practice. These findings reinforce the need for integrated, patient-centered therapeutic approaches.
