# Supplementary material for: Health and disability assessment in individuals with hearing loss: a longitudinal study using WHODAS 2.0
Source: Codas. 2026 Jul 31;38(5):e20250297. doi: 10.1590/2317-1782/e20250297en (PMC13440681; doi:10.1590/2317-1782/e20250297en)
Supplement: Translation

# Translation: Avaliação de saúde e incapacidade em indivíduos com perda auditiva: um estudo longitudinal utilizando o WHODAS 2.0

**DOI:** 10.1590/2317-1782/e20250297pt

**Authors:** Karen Melissa Gonzaga dos Santos, Rogéria Cristina Toscano Dias, Jade Louise Alves Macedo Padilha Silva, Gizele Francisco Ferreira do Nascimento

**Affiliations:** 1 Residência Multiprofissional no Cuidado à Saúde da Pessoa com Deficiência, Instituto de Ensino e Pesquisa Alberto Santos Dumont – ISD - Macaíba (RN), Brasil.; 2 Programa Associado de Pós-graduação em Fonoaudiologia – PPgFon, Universidade Federal do Rio Grande do Norte – UFRN - Natal (RN), Brasil.

**Keywords:** Audição, Perda Auditiva, Classificação Internacional de Funcionalidade, Incapacidade e Saúde, Aparelhos Auditivos, Reabilitação da Deficiência Auditiva

## Abstract

**Objetivo:**

Avaliar a autopercepção de saúde e deficiência de indivíduos com perda auditiva (PA) bilateral antes e após um ano de uso de aparelho de amplificação sonora individual (AASI), utilizando o World Health Organization Disability Assessment Schedule 2.0 (WHODAS 2.0).

**Método:**

Estudo longitudinal, observacional e analítico-descritivo, aprovado pelo comitê de ética em pesquisa sob o número de parecer 6.767.314 no qual foram avaliados 15 indivíduos diagnosticados com PA de grau leve a severo, em uso de AASI bilateralmente. O WHODAS 2.0 foi aplicado no momento da adaptação e reaplicado após um ano de uso. As médias dos escores nos seis domínios do instrumento (cognição, mobilidade, autocuidado, relações interpessoais, atividades laborais e participação) foram analisadas estatisticamente.

**Resultados:**

Observou-se redução da incapacidade após o uso do AASI, com redução média de 12% nos escores do WHODAS. Domínios como cognição, autocuidado e participação apresentaram diferença estatística.

**Conclusão:**

O WHODAS 2.0 mostrou-se eficaz para avaliar e monitorar a incapacidade e seu impacto na funcionalidade de indivíduos com PA, sendo sensível às mudanças promovidas pelo uso do AASI, apesar da ausência de significância estatística em algumas variáveis.

## INTRODUÇÃO

A perda auditiva (PA) é uma das condições sensoriais mais prevalentes e negligenciadas do século XXI, afetando mais de 430 milhões de pessoas em todo o mundo e representando uma das principais causas de incapacidade, especialmente entre adultos e idosos^(1)^. Estima-se que, a partir dos 60 anos, cerca de 20% dos indivíduos apresentem algum grau de PA, muitas vezes não diagnosticada ou não tratada de forma adequada^(2)^. O impacto da PA transcende os aspectos auditivos e compromete diretamente a qualidade de vida, a saúde mental e as interações sociais das pessoas acometidas^(3)^.

A reabilitação auditiva é considerada demanda urgente dos sistemas de saúde pública, devido ao contexto do envelhecimento populacional global, que acarreta um crescente número de indivíduos com PA em decorrência da presbiacusia. O uso do aparelho de amplificação sonora individual (AASI) é considerado uma das principais estratégias terapêuticas para auxiliar a percepção auditiva e proporcionar melhorias na comunicação. Entretanto, a eficácia do AASI vai além do ganho audiométrico, pois exerce uma influência em domínios da vida cotidiana, como autonomia, participação social, desempenho ocupacional e bem-estar emocional^(3)^.

Para que seja possível avaliar o impacto dessas questões na qualidade de vida do indivíduo, é necessário que seja adotado o modelo biopsicossocial, que compreende a saúde como um resultado da interação entre a condição biológica e os fatores contextuais, ambientais e pessoais^(4)^. Nesse panorama, a Classificação Internacional de Funcionalidade, Incapacidade e Saúde (CIF) traz o conceito de funcionalidade como um termo abrangente para os aspectos positivos da interação, enquanto a incapacidade reflete os aspectos negativos, como limitações e restrições à participação^5.^ No contexto da audiologia, a CIF permite que a funcionalidade seja entendida como um termo abrangente para os aspectos positivos da saúde auditiva, enquanto a incapacidade reflete os desafios enfrentados pelo indivíduo em suas atividades e participação social^(4)^.

Essa perspectiva biopsicossocial é essencial para que a reabilitação auditiva ultrapasse o ganho audiométrico e foque na redução das restrições de participação vividas pelo sujeito^(4,5)^. Para quantificar esses parâmetros de forma padronizada, o *World Health Organization Disability Assessment Schedule 2.0* (WHODAS 2.0) foi desenvolvido como uma ferramenta diretamente fundamentada nos conceitos e domínios da CIF, permitindo mensurar o nível de incapacidade percebida em seis domínios: cognição, mobilidade, autocuidado, relações interpessoais, atividades de vida e participação social^(5)^.

O WHODAS 2.0 tem sido amplamente utilizado em diversas populações, incluindo indivíduos com doenças crônicas, transtornos mentais, deficiências físicas e neurológicas, e vem demonstrando confiabilidade, validade e sensibilidade a mudanças ao longo do tempo^(6,7)^. Apesar disso, sua aplicação em pessoas com deficiência auditiva ainda é pouco explorada na literatura, sobretudo em estudos que avaliem o impacto da adaptação do AASI na percepção de incapacidade. Pesquisas recentes sugerem que o uso contínuo de dispositivos auditivos convencionais, como o AASI, pode não apenas melhorar a compreensão da fala, mas também reduzir barreiras na participação social, aumentar a autoconfiança e promover maior engajamento em atividades sociais e ocupacionais^(8)^.

Diante disso, se torna necessário avaliar como perfil de saúde e incapacidade é afetada pelo uso do AASI, a partir de ferramentas como o WHODAS 2.0, pois a mesma pode fornecer subsídios clínicos para o planejamento de intervenções centradas no indivíduo e permitir uma mensuração objetiva dos resultados da reabilitação auditiva sob perspectiva biopsicossocial, além de contribuir para a formulação de políticas públicas que reconheçam o impacto real da PA na vida das pessoas e garantam intervenções eficazes.

Assim, o presente estudo teve como objetivo avaliar a autopercepção de saúde e deficiência de indivíduos com diagnóstico de perda auditiva bilateral, usuários de AASI, antes e após um ano de uso do dispositivo, utilizando o WHODAS 2.0 como instrumento de medida.

## MÉTODO

Trata-se de um estudo longitudinal, observacional e analítico-descritivo, aprovado pelo Comitê de Ética em Pesquisa sob o número de Parecer 6.767.314. A casuística preliminar foi obtida por conveniência, a partir dos pacientes acompanhados pelo serviço de Saúde Auditiva do Instituto de Ensino e Pesquisa Alberto Santos Dumont, localizado na área rural do município de Macaíba, Rio Grande do Norte. Todos os indivíduos que aceitaram participar do estudo foram instruídos sobre os objetivos da pesquisa e assinaram o Termo de Consentimento Livre e Esclarecido (TCLE).

Foram incluídos 15 indivíduos com diagnóstico de PA bilateral, de grau leve a severo, indicados para uso de AASI. Os critérios de inclusão adotados foram: idade igual ou superior a 18 anos, ausência de outras deficiências sensoriais ou cognitivas que pudessem interferir na compreensão do instrumento e possuir disponibilidade para acompanhamento longitudinal. Foram excluídos os sujeitos com uso irregular do AASI ou com histórico de descontinuidade no acompanhamento audiológico.

A coleta de dados foi realizada de forma presencial com a aplicação do WHODAS 2.0, na versão de 36 itens, conduzida por fonoaudiólogas treinadas para a utilização do instrumento. A primeira aplicação foi realizada entre janeiro e dezembro de 2023, durante o processo inicial de adaptação ao AASI. A segunda aplicação ocorreu entre janeiro e dezembro de 2024, após aproximadamente 12 meses de uso regular do dispositivo. Além disso, por meio de análise de prontuário, foram coletadas informações de idade, sexo, tipo e grau da PA, nível de escolaridade e situação ocupacional dos participantes.

O WHODAS é um instrumento prático, desenvolvido pela OMS, baseado no conceito da CIF, com o objetivo de medir os níveis de saúde e incapacidade de forma padronizada, a partir da avaliação de seis domínios funcionais, sendo eles: D1 – cognição (compreensão e comunicação); D2 – mobilidade (movimentação e deslocamentos); D3 – autocuidado (higiene, vestuário e alimentação); D4 – relações interpessoais; D5 – atividades domésticas e laborais; e D6 – participação em atividades sociais e comunitárias. Cada item do instrumento é pontuado de 0 a 4, no qual “0” representa nenhuma dificuldade e “4” dificuldade extrema ou incapacidade. Os escores foram calculados por domínio e também em formato global (soma total e porcentagem de incapacidade funcional).

A análise estatística foi realizada no programa *Statistical Package for the Social Sciences* (SPSS), versão 25.0. Inicialmente, aplicaram-se testes de normalidade (Kolmogorov-Smirnov), seguidos de estatística descritiva para caracterização da amostra. Para análise comparativa entre os escores antes e após o uso do AASI, utilizou-se o teste t de *Student* para amostras pareadas. O teste de Homogeneidade Marginal foi utilizado para verificar alterações na classificação funcional (leve, moderada, grave). Também foram realizados testes de correlação de Spearman entre idade, grau de perda e escore funcional, bem como ANOVA one-way para análise de variância entre os grupos. O nível de significância adotado foi de p<0,05.

## RESULTADOS

A amostra foi composta por 15 participantes, sendo 11 homens (73,3%) e 4 mulheres (26,7%), com idade média de 63,8 anos (±11,08).

Em relação ao grau de PA, 2 participantes apresentaram PA leve (13,3%), 8 moderada (53,3%) e 5 severa (33,3%). Todos os participantes foram adaptados bilateralmente com AASI digitais, programados conforme os limiares auditivos tonais obtidos na avaliação audiológica convencional. A Tabela 1 apresenta os dados sociodemográficos e audiológicos.

**Tabela 1:** Caracterização da amostra (n = 15)

**VARIÁVEL**	**VALOR**
**Sexo - n (%)**	11 (73,3%)
Masculino	4 (26,7%)
Feminino	
**Idade - média ± DP**	63,8 ± 11,08
**Perda auditiva - n (%)**	
Leve	2 (13,3%)
Moderada	8 (53,3%)
Severa	5 (33,3%)
**Escolaridade - n (%)**	
Ensino Fundamental Incompleto	4 (26,67%)
Ensino Fundamental Completo	8 (53,33%)
Ensino Médio Incompleto	0 (0%)
Ensino Médio Completo	3 (20%)
**Situação Ocupacional - n (%)**	
Atividade remunerada	7 (46,7%)
Aposentados	7 (46,7%)
Não exerce atividade laboral	1 (6,6%)

**Legenda:** n = número de sujeitos; DP = desvio padrão

Quanto ao perfil socioeducacional, observou-se que 53,3% da amostra possuía ao menos o ensino fundamental completo, enquanto 26,7% possuíam o fundamental incompleto e 20% apresentavam o ensino médio completo. Em relação à situação ocupacional, houve uma distribuição igualitária entre indivíduos aposentados (46,7%) em comparação aos que exerciam algum tipo de atividade remunerada (46,7%), enquanto apenas um participante (6,6%) relatou não exercer atividade laboral no momento (Tabela 1).

Na avaliação inicial com o WHODAS 2.0, a média total dos escores foi de 1,87 (±0,65), correspondendo a uma média percentual de 36,2%. Após um ano de uso regular do AASI, observou-se uma redução significativa na média total, que passou a 1,21 (±0,29), com média percentual de 24,2%. Essa redução corresponde a uma melhora funcional média de 12%, indicando menor impacto das limitações de saúde sobre as atividades cotidianas.

A Tabela 2 apresenta a comparação detalhada das médias por domínio antes e após o uso do AASI. Os domínios de cognição (p=0,015), autocuidado (p=0,017), atividades de vida/trabalho (p=0,044) e participação social (p=0,013) mostraram diferença estatística entre os momentos avaliados. Os domínios de mobilidade (p=0,142) e relações interpessoais (p=0,304), embora com tendência de melhora, não atingiram significância estatística.

**Tabela 2:** Médias do WHODAS 2.0 no momento inicial de adaptação do AASI e após um ano de utilização (n = 15)

**VARIÁVEL**	**VALOR**	**VALOR**	***p valor***
	**baseline**	**após 1 ano**	
**WHODAS 2.0 - média ± DP**	1,87 ± 0,65	1,21 ± 0,29	0,014^*^
Domínio 1 - Cognitivo	1,94 ± 0,65	1,34 ± 0,44	0,015*
Domínio 2 - Mobilidade	1,93 ± 0,95	1,46 ± 0,63	0,142
Domínio 3 - Autocuidado	1,4 ± 0,51	1,03 ± 0,87	0,017*
Domínio 4 - Relações interpessoais	1,72 ± 0,77	1,52 ± 0,36	0,304
Domínio 5 - Atividades de trabalho/domésticas	1,64 ± 0,89	1,10 ± 0,18	0,044*
Domínio 6 - Participação	2,12 ± 0,78	1,46 ± 0,62	0,013*
**Porcentagem total - média ± DP**	36,2 ± 11,14	24,2 ± 5,8	0,017*
**Grau de deficiência - n (%)**			0,083
Leve	7 (46,7%)	11 (73,3%)	
Moderada	6 (40%)	4 (26,7%)	
Grave	2 (13,3%)	0 (0,0%)	

* p < 0,05

**Legenda:** WHODAS 2.0 = *World Health Organization Disability Assessment Schedule 2.0*; n = número de sujeitos; DP = desvio padrão

Além disso, observou-se uma mudança no perfil funcional dos participantes: o número de indivíduos com deficiência leve aumentou de 7 (46,7%) para 11 (73,3%), enquanto os casos moderados diminuíram de 6 (40%) para 4 (26,7%) e os casos graves deixaram de ser observados. Apesar da melhora clínica evidente, o teste de Homogeneidade Marginal não indicou diferença estatística (p=0,083), possivelmente devido ao tamanho amostral reduzido.

As análises de correlação de Spearman não revelaram associação significativa entre os escores funcionais e a idade (p=0,380) ou o grau de perda auditiva (p>0,05). O teste de ANOVA também não identificou variabilidade significativa nos escores do WHODAS em função dessas variáveis, sugerindo que a melhora funcional ocorreu de forma independente do perfil audiológico de base.

## DISCUSSÃO

Os resultados deste estudo demonstram que o uso contínuo do AASI promoveu uma melhora mensurável na funcionalidade de indivíduos com PA, evidenciada pela redução dos escores do WHODAS 2.0 após um ano de acompanhamento. Embora algumas análises não tenham alcançado significância estatística, os resultados apontam para efeitos clínicos importantes, sobretudo nos domínios de cognição, autocuidado, atividades e participação social, áreas fundamentais para a manutenção da autonomia e qualidade de vida.

Esses achados reforçam a literatura que evidencia o impacto funcional da PA em diferentes esferas da vida. A presença de déficits auditivos está associada ao declínio das funções cognitivas, maior risco de depressão, prejuízos na comunicação interpessoal, isolamento social e diminuição na capacidade de realizar atividades instrumentais do cotidiano. Nesse sentido, a amplificação auditiva, ao restaurar parcialmente a percepção sonora, tende a aliviar a sobrecarga cognitiva imposta pela escuta dificultosa e a favorecer a reintegração social do indivíduo^(3,8,9)^.

O domínio de participação social mostrou-se um dos mais beneficiados pelo uso do AASI, com redução significativa nos escores de limitação. A literatura sugere que a participação em atividades sociais e comunitárias é uma das primeiras áreas impactadas pela PA, muitas vezes devido à insegurança comunicativa e ao estigma associado ao uso de próteses auditivas^(10)^. Com o uso regular do AASI, os usuários tendem a recuperar parte da confiança e da habilidade de engajar-se em contextos sociais, o que pode gerar efeitos positivos sobre a saúde mental e a autopercepção funcional^(11-13)^.

Outro achado foi a melhora no domínio de cognição, o que corrobora investigações recentes que apontam uma relação bidirecional entre PA e declínio cognitivo^(12,13)^. A hipótese da “carga cognitiva aumentada” sugere que, na presença da PA, o cérebro precisa redirecionar recursos cognitivos para compensar a perda sensorial, em detrimento de outras funções como memória, atenção e planejamento^(14)^. O uso do AASI pode reduzir essa sobrecarga e preservar funções executivas, especialmente em adultos mais velhos.

Além das melhoras significativas já esperadas nos domínios de Cognição e Participação Social, este estudo também apontou uma redução estatisticamente significativa da incapacidade nos domínios de Autocuidado e Atividades de Vida. A melhora no domínio “Atividades de Vida”, está possivelmente atrelada à redução da carga cognitiva proporcionada pelo AASI. Uma vez que o esforço de escuta diminui, o indivíduo consegue alocar mais recursos atencionais para a execução de suas tarefas ocupacionais e diárias^(15)^. Já em relação a melhora significativa no Autocuidado, pode-se inferir que, com a reabilitação auditiva, existe uma melhora na percepção do ambiente e uma autoconfiança na comunicação, podendo refletir em um aumento da autonomia global do indivíduo, que passa a se sentir mais seguro em gerenciar suas atividades pessoais de forma independente^(16)^.

A caracterização da amostra evidenciou um perfil de escolaridade predominantemente composto por pessoas com baixo nível de escolaridade e situação ocupacional homogênea. Somado a isso, a maior proporção de participantes do sexo masculino reflete o perfil da demanda do serviço onde a coleta foi realizada, tratando-se de uma amostragem por conveniência. No modelo biopsicossocial, o gênero é um fator pessoal relevante que, juntamente com a escolaridade, pode influenciar a compreensão acerca da saúde, bem como a adesão ao tratamento e a própria percepção da incapacidade. Tais achados são relevantes, visto que o baixo acesso educacional reflete, em sua maioria, um fator que pode influenciar a percepção da incapacidade^(11,12)^.

Além disso, a situação ocupacional, refletida pelo grande número de indivíduos inseridos no mercado de trabalho, pode estar correlacionada com a melhora do domínio ‘'Atividades de Vida’', sugerindo um impacto direto da reabilitação auditiva na manutenção da produtividade. Por outro lado, o número de participantes aposentados, também pode estar contribuindo para a melhora nos domínios de '’Participação Social’’ e '’Cognição’', uma vez que o AASI tem um papel fundamental na promoção de um envelhecimento ativo e na qualidade de vida^(13,16)^.

A ausência de significância nos domínios de mobilidade e relações interpessoais pode ter múltiplas explicações. A mobilidade, apesar de poder ser influenciada indiretamente pela audição, via equilíbrio e orientação espacial, não é diretamente afetada pela amplificação sonora nos indivíduos sem comorbidades otoneurológicas. Já o domínio de relações interpessoais envolve múltiplos fatores contextuais, emocionais e culturais, cuja mudança pode exigir intervenções complementares ao uso do AASI, como suporte psicológico.

O WHODAS 2.0 mostrou-se uma ferramenta sensível para captar variações funcionais decorrentes da reabilitação auditiva, mesmo em uma amostra reduzida. Sua abordagem baseada na CIF permite uma visão integrada e centrada no usuário, alinhada aos princípios da prática fonoaudiológica atual^(17)^. Estudos prévios validaram sua aplicabilidade em idosos, pessoas com deficiência e doenças crônicas^(13)^ e, no atual estudo, foi observada a sua aplicabilidade em usuários de AASI.

No entanto, ressaltam-se as limitações do presente estudo, como o tamanho amostral reduzido, que limitou a viabilidade de maiores análises estatísticas, podendo ter contribuído para a ausência de significância em algumas comparações, o tipo de amostragem, realizada por conveniência, que resultou em um desequilíbrio entre os sexos, visto que gênero é considerado um fator pessoal relevante no modelo biopsicossocial. Adicionalmente, dados referentes à renda dos participantes não foram discutidos, limitando a análise entre diferentes perfis socioeconômicos. Sendo assim, a forma como a incapacidade foi percebida entre os grupos não pôde ser explorada.

Apesar disso, os resultados observados têm implicações práticas relevantes. O WHODAS 2.0 pode ser incorporado à rotina clínica como ferramenta de avaliação e acompanhamento longitudinal da funcionalidade em indivíduos com PA, auxiliando na definição de metas terapêuticas personalizadas e na avaliação do impacto da reabilitação auditiva sob múltiplas perspectivas.

## CONCLUSÃO

O presente estudo demonstrou que o uso contínuo do AASI está associado à melhora funcional em indivíduos com perda auditiva, especialmente nos domínios de cognição, autocuidado, atividades de vida e participação social. O WHODAS 2.0 mostrou-se eficaz para mensurar essas mudanças ao longo do tempo, reforçando seu valor como instrumento clínico de acompanhamento.

Apesar da ausência de significância estatística em algumas análises, observou-se uma tendência clínica positiva, destacando a importância da avaliação da funcionalidade na prática fonoaudiológica. Esses achados reforçam a necessidade de abordagens terapêuticas integradas e centradas no indivíduo.
